# Magnetite Nanoparticles Functionalized with RNases against Intracellular Infection of *Pseudomonas aeruginosa*

**DOI:** 10.3390/pharmaceutics12070631

**Published:** 2020-07-06

**Authors:** Nathaly Rangel-Muñoz, Alejandra Suarez-Arnedo, Raúl Anguita, Guillem Prats-Ejarque, Johann F. Osma, Carolina Muñoz-Camargo, Ester Boix, Juan C. Cruz, Vivian A. Salazar

**Affiliations:** 1 Department of Biomedical Engineering, Universidad de los Andes, Cra. 1E No. 19a-40, Bogotá 111711, Colombia; in.rangel@uniandes.edu.co (N.R.-M.); sa.suarez1912@uniandes.edu.co (A.S.-A.); c.munoz2016@uniandes.edu.co (C.M.-C.); 2 Department of Biochemistry and Molecular Biology, Faculty of Biosciences, Universitat Autònoma de Barcelona, 08193 Cerdanyola del Vallès, Spain; raul.anguita@e-campus.uab.cat (R.A.); Guillem.Prats.Ejarque@uab.cat (G.P.-E.); 3 Department of Electrical and Electronics Engineering, Universidad de los Andes, Cra. 1E No. 19a-40, Bogotá 111711, Colombia; jf.osma43@uniandes.edu.co

**Keywords:** *Pseudomonas aeruginosa*, ribonucleases, magnetite nanoparticles, antimicrobials

## Abstract

Current treatments against bacterial infections have severe limitations, mainly due to the emergence of resistance to conventional antibiotics. In the specific case of *Pseudomonas* *aeruginosa* strains, they have shown a number of resistance mechanisms to counter most antibiotics. Human secretory RNases from the RNase A superfamily are proteins involved in a wide variety of biological functions, including antimicrobial activity. The objective of this work was to explore the intracellular antimicrobial action of an RNase 3/1 hybrid protein that combines RNase 1 high catalytic and RNase 3 bactericidal activities. To achieve this, we immobilized the RNase 3/1 hybrid on Polyetheramine (PEA)-modified magnetite nanoparticles (MNPs). The obtained nanobioconjugates were tested in macrophage-derived THP-1 cells infected with *Pseudomonas aeruginosa* PAO1. The obtained results show high antimicrobial activity of the functionalized hybrid protein (MNP-RNase 3/1) against the intracellular growth of *P. aeruginosa* of the functionalized hybrid protein. Moreover, the immobilization of RNase 3/1 enhances its antimicrobial and cell-penetrating activities without generating any significant cell damage. Considering the observed antibacterial activity, the immobilization of the RNase A superfamily and derived proteins represents an innovative approach for the development of new strategies using nanoparticles to deliver antimicrobials that counteract *P. aeruginosa* intracellular infection.

## 1. Introduction

The rapid emergence of resistant bacteria is occurring worldwide, endangering the efficacy of antibiotics and turning into a public health threat that can compromise millions of lives [1]. The antibiotic resistance crisis has been attributed to the overuse and misuse of these medications, as well as a lack of new drug development approaches against the emergence of multi-drug resistance (MDR) strains [2]. Over the last decade, studies have focused on the search and evaluation of new biomolecules, such as peptides and proteins, with potent antimicrobial activities [3,4]. These emerging molecules have been reported to act either alone or in combination to potentiate their action against pathogens. Antimicrobial proteins and peptides (AMPs) are naturally-occurring and abundantly distributed in various organs, tissues, and body fluids as part of the human innate system [5,6]. Human ribonucleases (RNases) of the superfamily of RNase A are a vertebrate-specific family of proteins of structurally homologous proteins to the bovine pancreatic ribonuclease A, also known as RNase A; this is perhaps, the best-characterized of all known mammalian enzyme proteins [7]. In humans, eight functional RNases’ (named canonical RNases [8]) genes have been identified within a single chromosome. This enzyme family includes RNase 1, or pancreatic RNase, expressed in the pancreas as well as in other organs and tissues [9]. Among all the canonical RNases, RNase 1 has the highest catalytic activity and a remarkable activity against dsRNA involved in the nonspecific response to pathogenic RNA molecules [10,11]. Regarding antimicrobial activity, RNase 3 stands out for its high bactericidal action and the efficacy of an N-terminus-derived peptide on Gram-negative bacteria biofilms. This ability to remove biofilms of Gram-negative species can be explained by its specific cell agglutination and lipopolysaccharide-binding properties [12,13,14,15]. Besides, RNase 3 was proved effective against macrophage intracellular infection. Interestingly, we have demonstrated the high performance of RNase 3, not only in bacterial infection removal but also in internalization within macrophage cells and autophagy activation [16]. In this context, we designed an RNase 3/1 hybrid that combines the unique features of RNase 3 and the high catalytic activity and ribonuclease inhibitor affinity of RNase 1 and tested the construct in an in vitro experimental evolution test with *Acinetobacter baumannii*, where it demonstrated its ability to delay the acquisition of colistin resistance [15]. Antimicrobial RNases have been reported to be effective against biofilm-forming pathogens, such as *Mycobacterium tuberculosis*, *A. baumannii*, or *Pseudomonas aeruginosa* [12,13]. 

*P. aeruginosa* is classified as an opportunistic pathogen that has been commonly associated with deadly infections, such as pneumonia. Moreover, among others, *P. aeruginosa* is a serious threat to immunocompromised and cystic fibrosis (CF) patients [17,18]. Some of these common health problems have been aggravated due to the emergence of bacterial resistance to drugs. For this reason, *P. aeruginosa* has been listed among the pathogens for the prioritized search of new antimicrobials [19]. Additionally, *P. aeruginosa* can be internalized by macrophages and escape vacuole entrapment. Moreover, once internalized, *P. aeruginosa* fosters a phase of intracellular residence mediated by several adaptation mechanisms that help the pathogen avoiding phagocytosis and ultimately, promoting extracellular multiplication and replication in other host cells [20,21,22,23,24,25]. Many studies have demonstrated the potency of cell internalization of pharmacological treatments as a very effective route to tackle persistent *P. aeruginosa* infections [17,20,21,22,25,26]. In particular, one pharmacological avenue of interest is the delivery of engineered AMPs to improve their internalization capacity. Some of the engineering strategies include functionalization with cysteine-pentaglycyl, o-dithiobenzyl carbamate, (acyloxy)alkyl ester and polyethylene glycol (PEG) [27,28,29]. Additionally, an emerging route to increase penetration is provided by the widely available nanocarriers. In this regard, a number of studies have been dedicated to interfacing AMPs with nanomaterials, such as iron oxide, gold, silver and carbon nanoparticles, to enhance penetration significantly and consequently, bioavailability [28,30,31,32]. In the case of microbial control via delivery of the nanobioconjugates of peptide drugs, recent successful examples include Cathelicidin LL-37-carbon nanoparticles, Buforin II-MNPs and Ubiquicidin (29–41)-silver-gold nanoparticles [31,32,33,34,35]. One of the most attractive nanodelivery systems is magnetite nanoparticles (MNPs), mainly due to their magnetic properties, which allow them to be a reliable platform for magnetic targeting, as external magnetic fields can be applied to guide their transport, fate and improve their localization within target tissues [36]. Additionally, once delivered into a particular organ or tissue, magnetic fields can be subsequently applied to facilitate processes such as thermal energy release, endosomal escape or disrupting endothelial cell–cell junctions [36]. MNPs can be easily visualized with the aid of MRI and magnetic particle imaging (MPI) instruments, which facilitate the ease monitoring of their localization within organs, tissues and cells both in in vitro and in vivo [36,37]. MNPs exhibit high biocompatibility and antimicrobial abilities [33,34,38]. They also have been widely employed to immobilize enzymes, mainly due to the possibility of enhancing their catalytic performance [39], thermal and long term stability [40] and reusability [41,42,43].We recently developed cell-penetrating nanobioconjugates of MNPs by interfacing them with the AMP Buforin II (BUF-II) and the Outer Membrane Protein A (OmpA) of *Escherichia coli* [33,34]. The synthesized nanobioconjugates not only penetrated a number of mammalian cell lines but efficiently escaped endosomes. Moreover, they exhibited high biocompatibility, as evidenced by low cytotoxicity and reduced hemolytic tendency. To maintain the antimicrobial activity of BUF-II, we modified the MNPs with a polyetheramine (PEA) surface spacer. The polyetheramines contain oxygen moieties that are described in the literature as highly biocompatible. Additionally, these spacers have been reported as useful surface linkers because of the flexibility imparted by the polyether groups present in their backbones [44]. Besides, it has been reported that RNases have been immobilized on different types of supports, including magnetic microspheres [45], dextran nanogels [46], silica nanoparticles [47], and lipidoids [48]. The prepared immobilizates found application in the delivery of RNase A for cancer treatment [49] and RNase H for the detection of pathogenic bacterial DNA [50]. By taking advantage of the potent antimicrobial activity reported for hybrid RNase 3/1 [15], we designed the bionanoconjugate MNP-RNase 3/1.

This work is therefore dedicated to immobilizing the hybrid RNase 3/1 on magnetite nanoparticles to increase therapeutic efficacy against *P. aeruginosa* at the intracellular level. We synthesized and functionalized the nanoparticles to obtain the RNase 3/1 or RNase A nanobioconjugates. Then, they were characterized with the aid of light scattering, spectroscopy, and thermal techniques. Moreover, we evaluated their biocompatibility, cytotoxicity, cell-penetrating abilities, and the control of extracellular and intracellular infection by *P. aeruginosa*.

## 2. Materials and Methods 

### 2.1. Materials

Magnetic nanoparticle (MNP) synthesis and functionalization were conducted with 98% Iron (II) chloride tetrahydrate (PanReac AppliChem, Chicago, IL, USA) and 97% Iron (III) chloride tetrahydrate from (Sigma-Aldrich Co., Munich, Germany), 98% sodium hydroxide (NaOH), dimethylformamide (DMF) (PanReac AppliChem, Chicago, IL, USA), 25% tetramethylammonium hydroxide (TMAH), 98% (3-Aminopropyl) triethoxysilane (APTES), 98% *N*-hydroxysuccinimide (NHS), 98% *N*-[3-dimethylammino)-propyl]-N′-ethyl carbodiimide hydro-chloride (EDC), 99% dimethyl sulfoxide (DMSO), >95% Rhodamine B, and 25% glutaraldehyde (Sigma-Aldrich, Munich, Germany). Triton™ X-100 was purchased from (Sigma-Aldrich). Ribonucleic acid from baker’s yeast (*S. cerevisiae*) (Sigma-Aldrich, Munich, Germany) was assayed for catalytic analysis. Luria–Bertani (LB) for *P. aeruginosa* PAO1 (Sigma Aldrich, Munich, Germany) were used for the bacterial cultures. Jeffamine M-600 polyetheramine (PEA) (Huntsman, Salt Lake City, UT, USA) was used as a surface spacer and oxidized with the aid of KMnO_4_ (Sigma-Aldrich, Munich, Germany) to render free terminal carboxyl groups for further conjugation. DMEM, RPMI 1640 Medium, fetal bovine serum (FBS) and trypsin EDTA were obtained from Biowest (Barcelona, Spain). Penicillin/Streptomycin (P/S) was purchased from Lonza (Basel, Switzerland) and Gentamicin (80 mg/mL) were purchased from Genfar (Bogotá, Colombia). Phorbol 12-myristate 13-acetate (PMA) was used to induced monocyte THP-1 differentiation into macrophages (Santa Cruz Biotechnology, Dallas, TX, USA). Cytotoxicity and delivery were carried out in THP-1 (NCTC, #88081201) and Vero (ATCC^®^ CCL-81). Cell dyes—Vybrant™ DiO Cell-Labeling Solution, LysoTracker™ Green DND-26 and Hoechst 33342 Staining Dye Solution—were all purchased from Invitrogen (Carlsbad, CA, USA). Finally, the bacterium strain was *P. aeruginosa* (ATCC 15915). 

### 2.2. Synthesis of Magnetic Nanoparticles

A mixture of 1 g of FeCl_2_·4H_2_O and 2.71 g of FeCl_3_·6H_2_O was dissolved separately in 5 mL of distilled water to obtain 1 and 2 M solutions, respectively. Chloride solutions were then mixed and heated up until a homogeneous solution was obtained. When the solution reached 90 °C, 2 mL of 2% *v*/*v* solution of TMAH was added. At the same time, 1.6 g of NaOH were dissolved in 5 mL of Type I water (8 M solution), and this solution was also heated to 90 °C. The hot NaOH solution was finally dropped at 200 µL/min into the chloride mixture under vigorous stirring at 1500 rpm. The reaction was carried out for 1 h at 90 °C under continuous stirring at 1500 rpm. The obtained MNPs were washed at least 3 times with distilled water with the aid of a strong permanent magnet, and sonicated after each wash (2800 ultrasonic cleaner, Branson, MO, USA) for 15 min at 40 kHz frequency. The obtained MNPs size was measured via Dynamic Light Scattering (DLS) (Zeta-Sizer Nano-ZS, Malvern, UK).

### 2.3. Surface Functionalization 

For surface functionalization, 100 mg of magnetite were dissolved in distilled water and sonicated vigorously until complete homogenization. In total, 2 mL of 2% (*v*/*v*) solution of TMAH, 50 µL of 99% glacial acetic acid and 100 µL of 10% (*v*/*v*) APTES were added to resuspend the MNPs. The sample was then kept for 1 h at 60 °C and 250 rpm to carry out the chemical reaction. Finally, the sample was washed several times with 1.5% (*w*/*v*) NaCl saline solution and Type I water to remove excess reagents. 

### 2.4. Ribonuclease A and Ribonuclease 3/1 Immobilization 

An amount of 2 mL of 2.5% (*v*/*v*) solution of glutaraldehyde was added to 100 mg of the MNPs sample and the mixture was left under mechanical agitation at 250 rpm at room temperature. Next, 300 µL of oxidized PEA were added and left overnight at 250 rpm. Then, 25 mg of RNase protein diluted in distilled water was mixed with an aqueous solution of 5 mg of NHS and 5 mg of EDC as crosslinking agents at 30 °C. The sample was left at 250 rpm overnight to obtain the MNPs-RNase nanobioconjugates. Finally, the MNPs-RNase samples were washed with NaCl solution 1.5% (*w*/*v*) and distilled water to remove excess reagents, aided by a permanent magnet. The effective conjugation of the RNase on the MNPs was verified with the aid of Fourier-transform Infrared Spectroscopy (FTIR) (Bruker Alpha II FTIR Eco-ATR (Bruker, Ettlinglen, Germany) and Thermogravimetric Analysis (TGA) (TA Instruments, New Castle, DE, USA). The concentration of immobilized protein per mass of nanoparticles was quantified colorimetrically using a commercial BCA Protein Assay Kit [51] (Supplementary Materials Figure S1) (Sigma-Aldrich, Munich, Germany).

### 2.5. Labeling of MNPs-RNase Nanobioconjugates with Rhodamine B 

A mixture of 5 mg of EDC and 5 mg of NHS was mixed with 5 mL of distilled water under vigorous agitation at 210 rpm and 38 °C temperature for 10 min. Next, 3 mg of Rhodamine B was added to the mixture and vortexed to obtain a homogeneous solution. Finally, the Rhodamine B solution was added to 10 mg of MNPs-RNase covered with aluminum foil to protect from photobleaching and left under agitation and refrigerated overnight. The labeled sample was washed several times under sterile conditions with distilled water until no traces of Rhodamine B were detectable. 

### 2.6. Hemolysis Assay

Hemolytic activity of MNPs-RNase was quantified according to Cuellar et al. [33]. A blood sample was obtained from a healthy volunteer in vacutainer blood tubes with EDTA. The samples were obtained with the approval of the Ethical Committee at the Universidad de los Andes (minute number 928-2018). In total, 25 mL of blood was centrifuged at 1800 rpm for 5 min; after that, the plasma supernatant was removed and replaced with 0.9% (*w*/*v*) NaCl solution. This process was performed in triplicate and the last supernatant was replaced by PBS buffer at pH 7.4. Serial dilutions of MNPs and MNPs-RNase were prepared in concentrations from 100 µg/mL to 12.5 µg/mL. In total, 100 µL of the MNPs-RNase were mixed with 100 µL of diluted 1:10 red blood cells and incubated in a 96-well plate for 1 h at 37 °C. Triton X-100 and PBS 1× were used as positive and negative controls, respectively. 

### 2.7. Platelet Aggregation Test

As for the hemolysis assay, a blood sample from a healthy volunteer was obtained with vacuum blue collection tubes containing Na_3_C_6_H_5_O_7_ and centrifuged at 1000 rpm for 15 min until a transparent supernatant with platelets was obtained. Samples of MNPs, MNPs-RNase A and MNPs-RNase 3/1 were previously sonicated and suspended in PBS 10× solution and finally, transferred to a microplate well to form serial dilutions from 100 to 6.25 µg/mL in triplicate. A blank without any nanomaterials was included in the experimental set. Platelets were added to each sample and left to interact for 3 min at room temperature. Subsequently, samples were measured in a microplate spectrophotometer (Thermo Scientific™, Waltham, MA, USA) at 620 nm. Thrombin and epinephrine were used as high positive platelet aggregation controls and PBS as the negative control.

### 2.8. LDH Cytotoxicity Assay

The MNPs-RNase cytotoxicity was carried out on human monocytic THP-1 and African green monkey kidney Vero cells via lactate dehydrogenase (LDH) assay in 96-well cell culture flat-bottom plates [21]. Nanobioconjugates of MNPs-RNase A and 3/1 were serially diluted in RPMI or DMEM medium from 1 to 0.3 mg/mL and then, added to a cell density of 5 × 10^5^ cells/mL. Samples were incubated for 24 h. The positive control was cells treated with 10% Triton X-100, while the negative control was untreated cells. Then, samples were centrifuged at 1100 rpm for 5 min and 50 μL of supernatant was transferred to a 96-well plate, mixed with 50 μL of LDH reaction mixture and incubated for 30 min. Finally, absorbance was measured at 490 and 650 nm for reference.

### 2.9. Catalytic Evaluation of MNPs-RNases by Activity Staining Plates

To evaluate the catalytic activity of RNases after immobilization, the samples were analyzed by staining activity agarose plates with total RNA yeast used as substrate by following the protocol of Radomski et al. [24]. MNPs-RNases and protein samples were placed over a plate that contained 0.1% of yeast RNA, 1% agarose in 20 mM sodium acetate (pH 5.0) at 37 °C for 30 min. Proteins were assayed at a 400 ng concentration and MNPs-RNase at 5000 ng. The percentage of catalytic activity was measured as a ratio between the bright area and the dark background using ImageJ/Fiji^®^ (Madison, WI, USA). 

### 2.10. Bacterial Cell Viability Luminescent Assay 

Bacterial viability was assayed using the BacTiter-Glo™ microbial cell viability kit, which allows measuring the viability of cells by ATP quantification with an indirectly measured luminescence detection assay. Overnight bacterial culture was subcultured until it reached OD_600_ = 0.4. Subsequently, serial dilutions of ribonucleases and nanobioconjugates (from 500 to 15 µg/mL) were incubated with *P. aeruginosa* PAO1 (1.6 × 10^6^ cells) in 10 mM HEPES buffer (pH 7.5) for 4 h at 37 °C. After incubation, 50 μL of the BacTiter-Glo™ reagent was added to each well and incubated at room temperature for 15 min. Luminescence was read on a Victor3 plate reader (Perkin-Elmer, Waltham, MA, USA) with a 3 s integration time. Luminescence signal to viable cell counts correlation was checked for the untreated *P. aeruginosa* cell controls. MBC was defined as the lowest concentration of MNPs, protein or MNPs-protein that resulted of >90% reduction in the initial inoculum.

### 2.11. THP-1 Cell Culture and Macrophage Induction

Human THP-1 cell culture was maintained at 37 °C in 25 cm^2^ tissue culture flasks using RPMI-1640 medium with 10% FBS and 1% P/S. Cell viability was assessed by Trypan Blue counting. THP-1 cells were treated with phorbol 12-myristate 13-acetate (PMA; 100 nM; (Santa Cruz Biotechnology, Dallas, TX, USA) at 37 °C for at least 24 h to obtain PMA-differentiated THP-1 macrophages. They were then cultured in RPMI-1640 at 37 °C in a humidified 5% CO_2_ incubator. Cells were allowed to rest for 24 h before infection and exposure to treatments.

### 2.12. Macrophage Infection 

*P. aeruginosa* PAO1 was cultured at 37 °C in a LB bacterial broth overnight. Prior to THP-1 cell infection, bacterial cells were incubated in fresh LB medium to an OD_600_ of 0.2, as previously established [52]. Cell culture concentration was adjusted to 3 × 10^5^ cells/mL and infected with *P. aeruginosa* at a MOI of 10 resuspended in RPMI-1640 medium in 96-well plates. Time of infection was 3 h followed by 3 washes with fresh PBS. Gentamicin at 400 µg/mL was added to the cell culture to remove the presence of residual extracellular *P. aeruginosa.* Finally, infected macrophages were washed and treated by MNPs-RNase A and MNPs-RNase 3/1 treatment in concentrations ranking from 500 to 7 μg/mL for 24 h. After the treatment exposure, cells were lysed with distillated water and seeded in a Luria–Bertani agar plate. Colony-forming units (CFU) were counted after 24 h to calculate the Intracellular Minimum Inhibitory Concentration (IMIC).

### 2.13. Cell Translocation of Nanobioconjugates

Cell translocation of MNPs-RNase A and MNPs-RNase 3/1 was performed on monocytic cell line derived from acute monocytic leukemia (THP-1) and induced macrophages. MNPs-RNase were previously labeled with Rhodamine B for observation under the confocal microscope. First, THP-1 cells were incubated with each MNPs-RNase A or MNPs-RNase 3/1 treatment at the intracellular MIC concentration of the MNPs-RNase 3/1 for 1 h. Then, the cells were washed twice with PBS 1× to prevent the interference of the non-penetrating bionanoconjugates. RPMI was prepared with Hoechst 33342 (1:1000 dilution) and Lysotracker (1:10,000 dilution) and then, 1 mL was added to cells. Samples were later imaged under a confocal laser scanning microscope (CLSM) with a PlanApo 60×, 1.35 NA oil-immersion objective with Ex/Em 358/461 nm (Hoechst 33342), Ex/Em 504/511 nm (Lysotracker green), and Ex/Em 577-556/590-580 (Rhodamine B). Images were collected at different positions throughout the culture depth to ensure a plot z and y-stack scan. For each z-stack image of THP-1 cells, we collected about 20 images along the depth of the region of interest. ImageJ/Fiji^®^ were used for image analysis.

### 2.14. Internalization Percent Determination

Internalization percentage in THP-1 cells was calculated by recording the fluorescent intensity of labeled nanobioconjugates of MNPs-RNase A and MNPs-3/1 after 1 h of exposure. This was accomplished by seeding 3 × 10^4^ cells/well in 96-well black plates followed by exposure to 100, 50, 25, 12.5, and 6.25 µg/mL of the labeled nanobioconjugates suspended in phenol-red-free RPMI medium. Fluorescence intensity of labeled nanobioconjugates was recorded in a spectrofluorometer (FluoroMax-4, Horiba, Japan) with Ex/Em wavelengths at 523/625 nm, respectively. Autofluorescence of cells and unlabeled nanobioconjugates were used as blanks. 

### 2.15. Infection of Macrophages with Stained P. aeruginosa

We prepared macrophages from THP-1 cells, as described above. Prior to infection, we stained *P. aeruginosa* cells with 1:200 concentration of DIO dye to facilitate their intracellular observation [53]. Macrophages were infected as described previously and left for 25 min to allow *P. aeruginosa* phagocytosis [21]. After exposure, macrophages were washed with PBS and we added 1:1000 ratio of Hoechst 33342 in RPMI supplemented with 400 µg/mL of gentamicin for nuclei staining. Macrophages were also exposed to labeled nanobioconjugates of MNPs-RNase A and MNPs-RNase 3/1 at the intracellular MIC (IMIC) for 1 h. Finally, the cells were washed with PBS before observation by confocal microscopy at the corresponding Ex/Em wavelengths for stained nuclei and nanobioconjugates (see above). In the case of labeled *P. aeruginosa*, imaging was conducted at Ex/Em484/501 nm. 

### 2.16. Statistical Analysis

The Student’s *t-*test and two-way ANOVA were applied to evaluate the significance of the differences between treatments. The results were plotted and analyzed using OriginPro 2018^®^ (OriginLab Corporation, Wellesley, MA, USA).

## 3. Results

### 3.1. Ribonuclease A and Ribonuclease 3/1 Immobilization on Magnetite Nanoparticles (MNPs)

Recently, we prepared bionanoconjugates of MNPs interfaced with antimicrobial and translocating molecules [33,34]. Here, we followed the immobilization protocol that led to superior antibacterial activity for the AMP Buforin II. According to this approach, immobilization proceeded on MNPs modified with an oxidized PEA surface spacer [33]. Consequently, we conducted immobilization of RNase 3/1 and RNase A on PEA modified MNPs to preserve their biological function.

**Figure 1:**
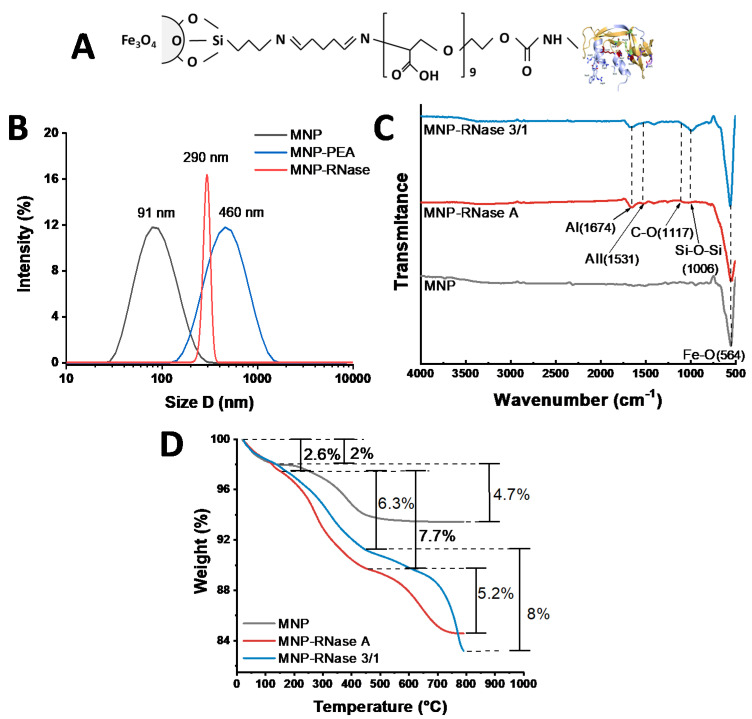
(**A**) Schematic representation of the chemical structure of the MNP-RNase bionanoconjugate. (**B**) DLS histogram for the size distribution of magnetite nanoparticles before (gray), PEA coated NPs (blue) and after immobilization MNP-RNase (red) nanobioconjugates. (**C**) FTIR spectra of bare magnetite (gray), MNP-RNase A nanobioconjugates (red), and magnetite-RNase 3/1 nanobioconjugates (blue). AI—Amide I and AII—Amide II. (**D**) TGA thermograms of magnetite (gray) and MNP-RNase A nanobioconjugates (red), and magnetite-RNase 3/1 nanobioconjugates (blue). The first weight loss steps (2.0%) represent the dehydration of the samples. Second weight loss steps (4.7%, 7.7%, and 6.3%) correspond to physically adsorbed organic solvents. The final weight loss steps (5.2% and 8.0%) are attributed to the detachment of RNase A and RNase 3/1 from the surface of magnetite nanoparticles.

The bionanoconjugate obtained after immobilization of RNases on PEA-modified MNPs is shown schematically in Figure 1A. Figure 1B shows the hydrodynamic diameter (HD) distribution of bare MNPs in contrast with the MNP-RNases bionanoconjugates as determined by DLS. Initially, bare MNPs approached a mean hydrodynamic diameter of 91 nm with a polydispersity index (PI) of 49%. After PEA coating, HD was measured at 460 nm with a %PI of 23 and finally, after conjugation of RNase 3/1, the hydrodynamic diameter increased to 290 nm with a PI of 22%. Those values of HD and PI% for MNP prior and after immobilization are comparable to those obtained in our previous work [33]. As shown in Figure 1B, MNP-RNase presents higher hydrodynamic diameters, thereby reproducing our previous results after conjugation of Buforin-II on PEA-modified MNPs [33]. The increase in hydrodynamic diameter is most likely due to aggregation phenomena during the immobilization of RNases [54]. This undesired effect most likely takes place during the washing steps, where 1.5% NaCl is added to the suspension to neutralize superficial charges and induce precipitation of the nanobioconjugates. Immobilization is also likely to expose hydrophobic moieties of the proteins, which exhibit a marked tendency for interaction and ultimately, promote clustering. To overcome this issue, it was necessary to perform multiple washes with ultrapure water and prolonged sonication periods. 

Immobilization of both RNases was verified by FTIR and TGA analyses. Figure 1C shows the spectra for bare magnetite, MNPs-RNase A and MNPs-RNase 3/1 bionanoconjugates. In the case of bare magnetite, the spectrum exhibited an absorption band at around 564 cm^−1^, which can be attributed to the Fe-O bond of iron oxide and is present in all conjugation steps [55]. Observation of the Si-O-Si stretching vibration at about 1006 cm^−1^ confirms the presence of APTES in the MNP–AP complex. Conjugation of PEA on silanized magnetite (MNP-AP-PEA) was confirmed by an absorption peak at 1670 cm^−1^ for the presence of C=O stretching in the backbone of the polymer [56]. Finally, conjugation of the RNases was verified by the presence of the amide I band at 1674 cm^−1^ and the amide II band at 1531 cm^−1^, which can be related to the secondary structure of the proteins [57].

Conjugation efficiency of RNase A and RNase 3/1 on the surface of MNP was estimated via TGA (Figure 1D). MNP and MNP-RNases exhibited an initial weight loss of about 2.0%, mainly due to dehydration of the samples. Bare magnetite had a second weight loss of 4.7%, while the MNPs-RNase A and MNPs-RNase 3/1 nanobioconjugates were 7.7% and 6.3%, respectively. These losses are attributed to the physically adsorbed organic compound residues derived from the synthesis and functionalization processes. Finally, the detachment of the RNases from the magnetite was estimated with a final weight loss step of 5.2% for MNPs-RNase A and close to 8.0% for MNPs-RNase 3/1. These weight losses agree well with those found in our previous works [33,34]. Further evidence of the conjugation efficiencies was provided by a BCA test (Figure S1), where the weight ratio between the MNPs-RNase bionanoconjugate and the free RNase protein was about 9:2 (2.277 µg per mg of MNP) for the RNase 3/1 and 4:1 (i.e., 2.319 µg per mg of MNP) for RNase A. This allowed us to estimate that 1 mg/mL of nanobioconjugates correspond to 15.52 µM for RNase A and 16.62 µM for RNase 3/1, respectively.

### 3.2. Catalytic Activity Evaluation of MNPs-RNases

**Figure 2:**
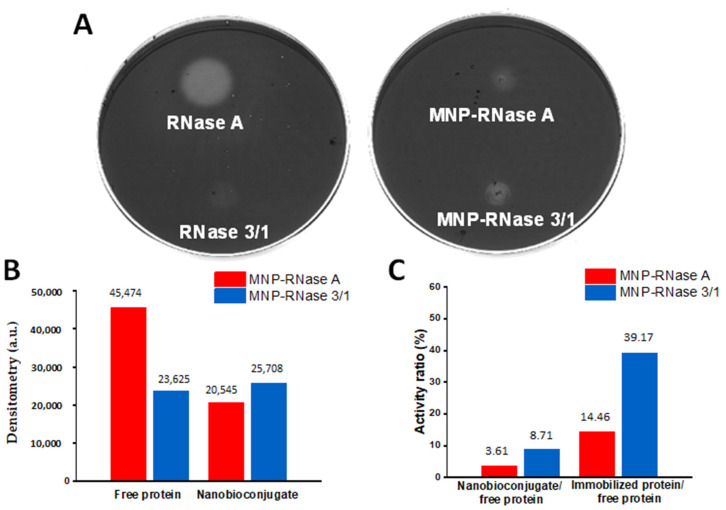
(**A**) Catalytic activity toward the total RNase of yeast. Light areas correspond to the sites of inoculation of the samples. The assay was conducted by depositing 5 µg of MNPs RNases (1.159 µM or MNPs-RNase A and 1.138 µM for MNPs-RNase 3/1) and 400 ng of free proteins and subsequently, incubating for 30 min. Images were collected with the aid of the Quantity One software^®^ (Bio-Rad). (**B**) Densitometric analysis of the light areas for native and immobilized proteins using ImageJ/Fiji^®^. (**C**) Activity ratio of nanobioconjugates compared to free proteins and activity ratio of immobilized compared to free proteins. The activity ratio between immobilized and free proteins was normalized by the amount of protein estimated via BCA.

The two obtained nanobioconjugates showed reduced catalytic activity when compared with the free proteins. This was evidenced after incubation for 30 min of 5 µg of MNPs RNases and 400 ng of native proteins (ratio 1:12 protein: bionanoconjugate; micromolar concentration of immobilized protein 1.16 and 1.14 for MNPs-RNase A and MNPs-RNase 3/1, respectively) on agarose enriched in yeast RNA. A quantitative analysis of Figure 2A,B shows that, when compared with the free proteins, the catalytic activity after immobilization was reduced by about 85.5% and 60.8% for RNase A and RNase 3/1, respectively (Figure 2C). We hypothesize that the loss of catalytic activity upon immobilization might be due to surface-induced conformational changes responsible for detrimentally altering the active sites, as reported elsewhere [58]. This notion should be verified by looking at possible secondary and tertiary structural changes with the aid of high-resolution biophysical techniques.

### 3.3. Biocompatibility Assessment

**Figure 3:**
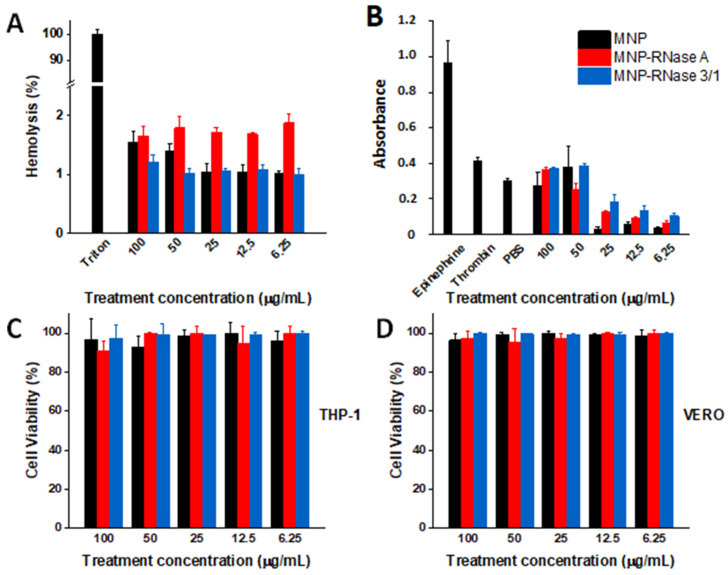
MNPs and both MNP-RNases exhibit high biocompatibility. (**A**) Assessment of the hemolytic effect of MNPs and MNPs-RNase nanobioconjugates. In all cases, hemolysis was below 5%, therefore complying with the ISO 10993 standard. Triton X-100 was used as a positive control. (**B**) Platelet aggregation for MNPs and MNPs-RNase nanobioconjugates. Platelet aggregation caused by MNPs and MNPs-RNase nanobioconjugates compared to Epinephrine and Thrombin. PBS was used as the negative control. For concentrations of 25 µg/mL and below, MNPs and both MNPs-RNases exhibited a low platelet aggregation tendency. (**C**) Cytotoxicity of MNPs and MNPs-RNase nanobioconjugates on THP-1 cells and (**D**) Vero cells.

To determine the potential as an antimicrobial treatment, the obtained magnetite-RNase nanobioconjugates need to be biocompatible. For this purpose, we followed the ISO 10993 standard to evaluate their hemolytic, platelet aggregation, and cytotoxic effect. Figure 3A shows hemolysis levels remained below 5% for all concentrations tested (100 to 6.26 µg/mL), thereby complying with the ISO standard. Figure 3B shows that for concentrations of 25 µg/mL and below, MNPs and both MNPs-RNases exhibited a low platelet aggregation tendency, as evidenced by levels below those of PBS, used as a negative control. For 50 and 100 µg/mL, the aggregation levels approached those of thrombin but were only half of those obtained with epinephrine, which is a strong aggregating agent. When compared with bare nanoparticles, the presence of the immobilized RNases appears not to have a further impact on aggregation. The intermediate levels of aggregation found here for the nanobioconjugates have been reported previously for other nanomaterials [59,60]. Of initial concern for this study was the potential cytotoxicity of RNases [61]. However, recent studies demonstrated that the modified RNase 3/1 protein showed low cytotoxicity for concentrations up to 200 μM [15]. Cytotoxicity assays were conducted for two model cell lines, i.e., THP-1 and Vero. The THP-1 monocytes were used instead of macrophages due to the possible interference of the differentiating PMA (phorbol myristate acetate) molecule with the LDH reagents. As a result, the presence of this compound could potentially lead to biased results [62,63,64]. Figure 3C,D show cell viability results for the MNPs-RNases on THP-1 and Vero cell lines, respectively. Our findings confirm that even at the highest concentration evaluated (i.e., 100 µg/mL), cell viability for both MNPs-RNase nanobioconjugates remains above 90% after 24 h. Recent studies have reported similar results for RNase A immobilized on different materials at concentrations lower than those evaluated here [46,65]. Taken together, these results suggest that the obtained nanobioconjugates are highly biocompatible, and therefore, have the potential to be considered for further in vivo studies.

### 3.4. Antibacterial Activity of MNPs-RNases Toward Extracellular and Intracellular P. aeruginosa

**Figure 4:**
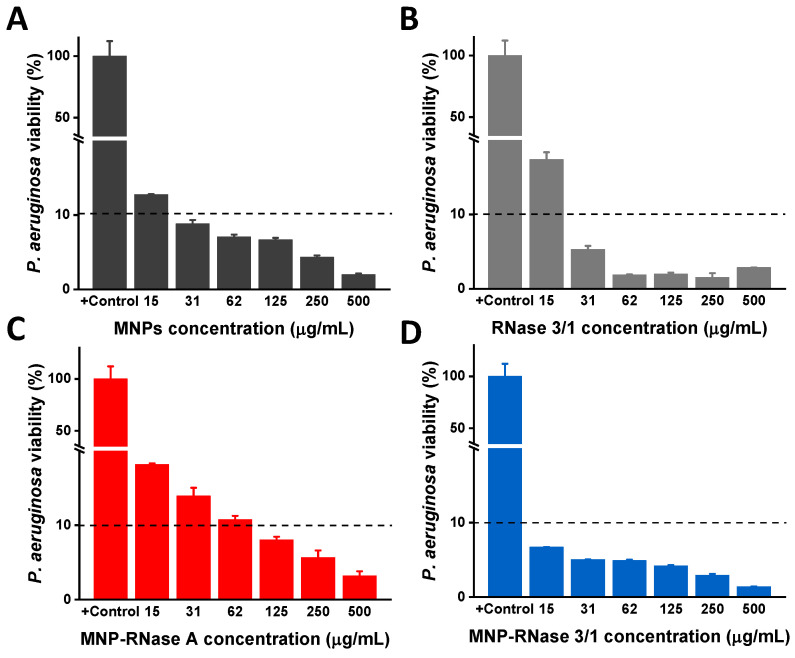
Antibacterial activity of free RNase 3/1 and nanobioconjugates of RNase 3/1 and A against *P. aeruginosa*. Each sample was analyzed after 4 h of incubation in 10 mM HEPES at pH 7.4. The negative control was untreated buffer. (**A**) Antibacterial activity of bare MNPs for concentrations ranging from 15 to 500 µg/mL. (**B**) Antibacterial activity of free Ribonuclease 3/1 for concentrations ranging from 15 to 500 µg/mL. (**C**) Antibacterial activity MNP-RNase A and (**D**) MNP-RNase 3/1 nanobioconjugates for concentrations ranging from 15 to 500 µg/mL. The horizontal black dashed line represents 90% bacterial viability.

We evaluated the antimicrobial efficiency of free and immobilized proteins. Antibacterial activity was determined by monitoring the cell viability of *P. aeruginosa* using the BacTiter-Glo™ luminescent kit. Using the coupled luciferin/luciferase assay, we quantified the ATP levels to estimate the number of viable bacterial cells in the culture. Free proteins and nanobioconjugates were assayed by serial dilutions starting from an initial concentration of 500 µg/mL. Viability of *P. aeruginosa* was established by measuring ATP and kanamycin was used as a positive control. The results indicated that bacteria viability decreased as a function of the bionanoconjugate concentration. As a remarkable finding, we detected antimicrobial activity when the bacterial cells were exposed to bare MNPs (Figure 4A), supporting similar studies [66]. Furthermore, free proteins exhibited an observable antimicrobial activity with a MBC_90_ = 31 µg/mL for RNase 3/1 (Figure 4B) and no significant inhibitory activity for RNase A (data not shown). Nonetheless, the activity was below that observed for the MNPs-RNase nanobioconjugates, which showed a high antimicrobial activity against *P. aeruginosa* (Figure 4C,D). In contrast, concentration of MNPs-RNase 3/1 of 15 µg/mL led to a significant reduction in viability of *P. aeruginosa* of ≥ 90% (10% of bacterial viability indicated by the dashed line). For MNPs-RNase A, a reduction in bacterial viability up to 90% was achieved at 125 µg/mL, as shown in Figure 4C. This result is somewhat surprising as RNase A is a direct ortholog of human RNase 1 that exhibits no antibacterial activity by itself [67,68,69]. These unexpected results must be likely related to the effect of MNPs in nanobioconjugates, as indicated above. Nevertheless, the antimicrobial effect of MNPs and MNP-RNase A was much lower in comparison to the results achieved by the MNP-RNase 3/1 bionanoconjugate (which showed a reduction by more than 90% in bacterial viability at 15 µg/mL). These results demonstrated that immobilization of RNases on MNPs, and in particular of the RNase 3/1 hybrid, is a suitable strategy to reduce *P. aeruginosa* viability.

Furthermore, although there is only 22.77 ± 0.01 (%weight) of RNase 3/1 immobilized on the nanoparticles, the obtained antibacterial activity is better than that of free ribonuclease. This represents an important advantage from the profit potential viewpoint, as less of the bioactive component is required for the same product performance. These results are consistent with findings reported in some previous studies, according to which, magnetite nanoparticles exhibit antimicrobial activity on their own [70,71] or are capable of improving that of the conjugated cargo [66,72].

**Figure 5:**
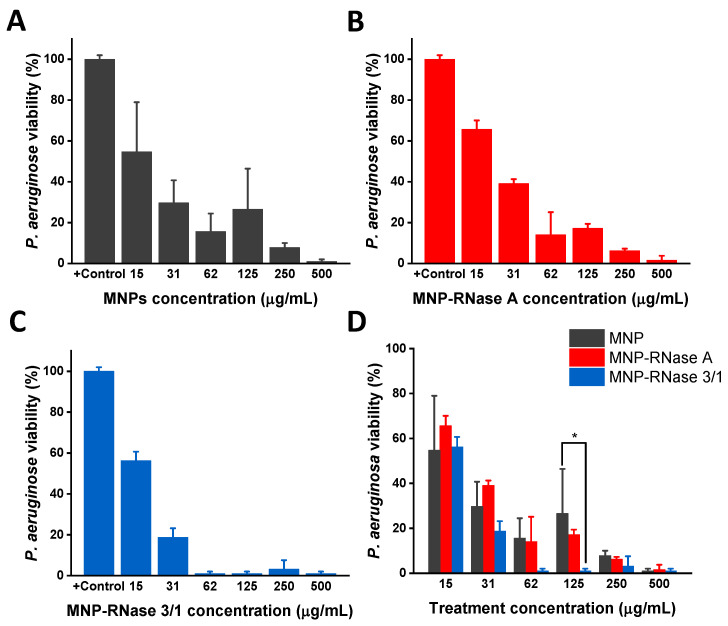
Minimum intracellular inhibitory concentration against *P. aeruginosa* in differentiated macrophages of (**A**) MNPs, (**B**) MNPs-RNase A and (**C**) MNPs-RNase 3/1 nanobioconjugates. Differentiated THP-1 cells were exposed at a multiplicity of infection (MOI) of 10:1. Posteriorly, the cells were incubated for 3h, washed and maintained in a fresh medium supplemented with gentamicin. (**D**) Significant differences between MNPs and MNP-RNase A or MNP-RNase 3/1 were estimated with the two-way ANOVA and Dunnett tests. * *p*-value < 0.05.

Once the extracellular activity of nanobioconjugates was determined, we analyzed their intracellular activity in macrophages infected with *P. aeruginosa*. Figure 5 shows the intracellular antimicrobial activity of nanobioconjugates on infected macrophages. The data were normalized with respect to the positive control, which consisted of infected cells without exposure to nanobioconjugates. At concentrations of 62.5 μg/mL of the MNP-RNase3/1, we achieved a reduction in *P. aeruginosa* growth above 90%. In contrast, we needed a concentration above 500 μg/mL for both MNPs and MNP-RNase A to reduce completely the intracellular growth of *P. aeruginosa*. Figure 5D demonstrates the potent antimicrobial activity effect of MNP-RNase 3/1 compared with bare MNPs and MNP-RNase A for concentrations above 62 μg/mL, which we considered the effective treatment concentration. A statistically significant difference was even detected for MNP-RNase 3/1 compared with bare MNPs, at a concentration of 125 μg/mL. In contrast to extracellular antimicrobial activity, the same level of intracellular reduction observed at a concentration of 62 μg/mL of bionanoconjugate (1.04 μM of total immobilized protein) required a higher concentration of the MNP-RNase 3/1 nanobioconjugates. Similar results were also obtained with MNPs-RNase A nanobioconjugates and MNPs at concentrations above 250 μg/mL.

### 3.5. Translocation of Nanobioconjugates and Intracellular Colocalization

**Figure 6:**
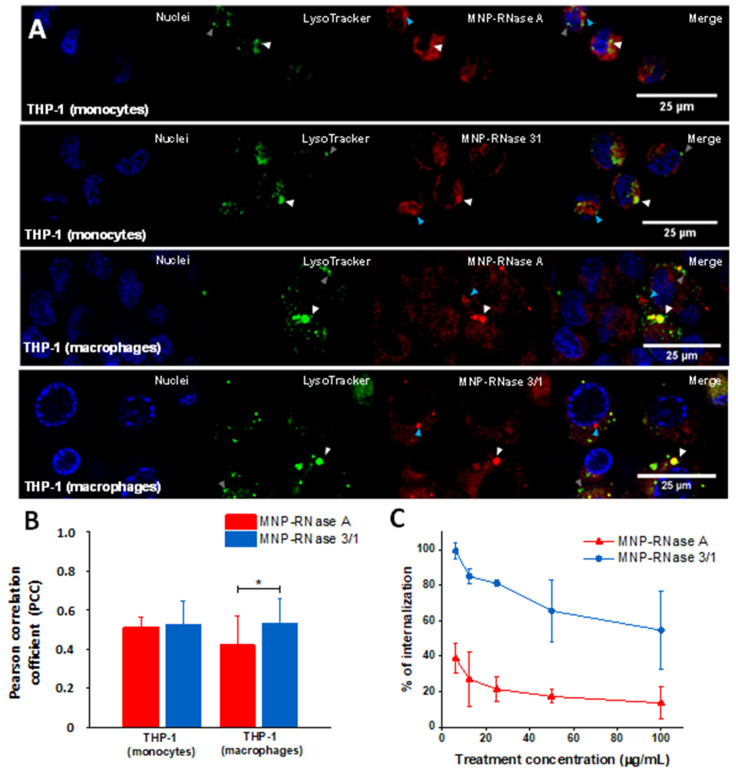
(**A**) Confocal microscopy of MNPs-RNase uptake into THP-1 and induced-macrophage cells. Both cell types were stained with Hoechst (blue) to visualize the nuclei. The nanobioconjugates were labeled with Rhodamine B (red), while lysosomes were stained with LysoTracker (green). White triangles indicate lysosomes, the blue ones pointed to bionanoconjugate accumulation outside lysosomal compartments and the gray ones represent empty lysosomal compartments. (**B**) Colocalization ratio was determined by image processing via the open access software Image J/Fiji^®^ (*n* = 20 fields with at least 5 cells, * *p*-value < 0.05). (**C**) Internalization percentage of MNPs-RNase A and 3/1 nanobioconjugates in THP-1 cells. At low concentrations of nanobioconjugates, uptake by the cells reaches between 40% and 100%. However, increasing the concentration saturates the cells and consequently, the nanobioconjugates uptake reduces to about 10 to 55%.

To study the translocation and internalization of the MNPs-RNase nanobioconjugates, we exposed both THP-1 monocytes and macrophage-induced cells to Rhodamine B-labeled MNPs-RNase A and MNPs-RNase 3/1. Assays were performed at a concentration of 62.5 μg/mL, which corresponds to about a 90% effective intracellular treatment concentration for MNP-RNase 3/1 (14.28 μg/mL or 1.04 μM of immobilized protein). In addition, the cells were stained with LysoTracker green to identify lysosomal compartments formed after the treatment with MNP-RNases. According to Figure 6A, nanobioconjugates labeled with Rhodamine B (red) are able to bypass cell membranes and distribute within the cytosol. Colocalizations of about 50% (as determined by the Pearson Correlation Coefficient—PCC) with Lysotracker for both cell types indicate that about half of the delivered MNPs-RNase A and MNPs-RNase 3/1 nanobioconjugates tend to accumulate in lysosomal compartments (see white arrows in Figure 6A). This is also summarized in Figure 6B. Interestingly, the MNP-RNase A nanobioconjugates showed a lower colocalization in macrophages in contrast with the colocalization determined in monocytes, which indicates a better ability to escape endosomes. Besides, it seems that the intracellular distribution of the nanobioconjugates varies depending on the cell type, with THP-1 monocytes showing a more homogeneous distribution. This was evidenced by evenly distributed intensity signals of Rhodamine B in THP-1 cells (see Rhodamine channel in Figure 6A). In contrast, for the macrophages, the obtained signal shows some clustering. However, as indicated by the blue arrows in Figure 6A, this clustering occurs in organelle compartments where no evident colocalization was observed. These results agree well with those reported by Luciani et al. [73], where macrophages showed higher endocytic activity than monocytes, despite similar uptake values of magnetite nanoparticles. This provides further evidence on the notion of the dependency of cell type on the intracellular distribution. Importantly, a number of lysosomal compartments appear empty, as evidenced by the absence of colocalization with Lysotracker (see gray arrows in Figure 6A). Finally, Figure 6C shows that after 1 h of incubation, cell internalization of the MNP-RNase 3/1 and MNP-RNase A nanobioconjugates approached 60% and 20%, respectively. This time was selected as it matched the one used for the hemolytic, cytotoxicity, platelet aggregation, and delivery tests.

**Figure 7:**
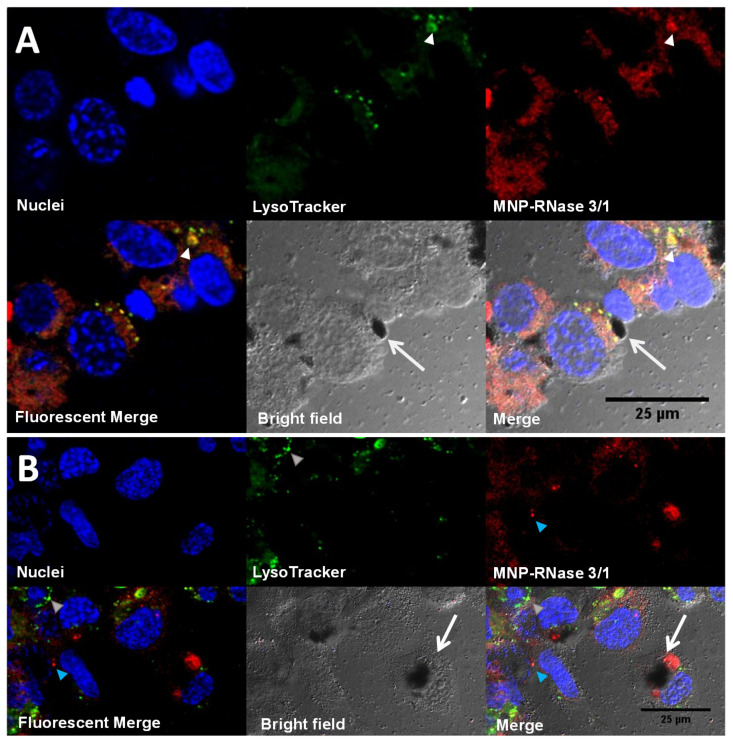
Intracellular distribution of nanobioconjugates in macrophages. (**A**) Macrophages with high accumulation of MNP-RNase 3/1 nanobioconjugates into lysosomal compartments (colocalization of 71 ± 8.1%, *n* = 5 cells in one visual field). The white triangles point to nanobioconjugates trapped in lysosomal compartments. The white arrows point to a nanoparticle cluster outside the cells. (**B**) Macrophages with a lower accumulation of MNP-RNase 3/1 nanobioconjugates into lysosomes (colocalization of 20 ± 10.6%, *n* = 5 cells in one visual field). The blue triangle points to accumulation of nanobioconjugates in compartments other than lysosomes. The white arrows point to an internalized large nanoparticle cluster.

Cellular uptake appeared higher for MNP-RNase 3/1 nanobioconjugates when compared with those of RNase A. This might be explained by the enhanced tendency of RNase 3/1 to interact with the biological membranes due to the incorporation in RNase 1 scaffold structure of RNase 3 singular regions (Prats-Ejarque, in preparation; [15]) [74,75]. Figure 7 shows the internalization and intracellular distribution of the MNP-RNase nanobioconjugates in macrophages. Figure 7A shows that the MNP-RNase 3/1 nanobioconjugates tend to accumulate in lysosomes (see white triangles in Figure 7A) as evidenced by a punctuated distribution along most of the cytosol. Due to the diameter of our nanobioconjugates of around 100 nm, we hypothesize that internalization is likely to have proceeded by a multipathway mechanism that involves clathrin-mediated and/or caveolin-mediated endocytosis [76,77]. Moreover, it has been reported that native RNase 3 tends to accumulate in the specialized primary lysosomes of eosinophil cells, which provides further evidence of the tendency of our MNPs-RNase 3/1 nanobioconjugates to remain trapped in the lysosomal compartments of macrophages [78]. In contrast, other studies conducted by our research group demonstrated that functionalized MNPs without immobilized protein have lower colocalization with lysosomal compartments in comparison to MNPs-RNases [79]. Figure 7B exemplifies how large nanobioconjugate clusters can be also internalized and accumulate without altering cell morphology. We believe that the pendant groups of PEA facilitate cell penetration, as has been previously described by us and others [33,79]. In particular, the intermingling with glycosaminoglycans and phospholipids in the membrane appear to be involved in bypassing the bilayer [80]. The internalization mechanisms associated with this type of polymeric cell penetrators appear to be endocytic and proceed by multiple pathways [81]. These results suggest that uptake is likely to occur by different mechanisms, a notion that is reinforced by the ability of macrophages to internalize exogenous material by phagocytosis [82]. Taken together, these results suggest that internalization takes place by different mechanisms. Additionally, because colocalization approaches 50% on average, some of the nanobioconjugates can escape such compartments. Further studies will be required to elucidate in more detail the involved internalization mechanisms.

### 3.6. Analysis of Infected Macrophages with P. aeruginosa and Exposed to MNPs-RNase

**Figure 8:**
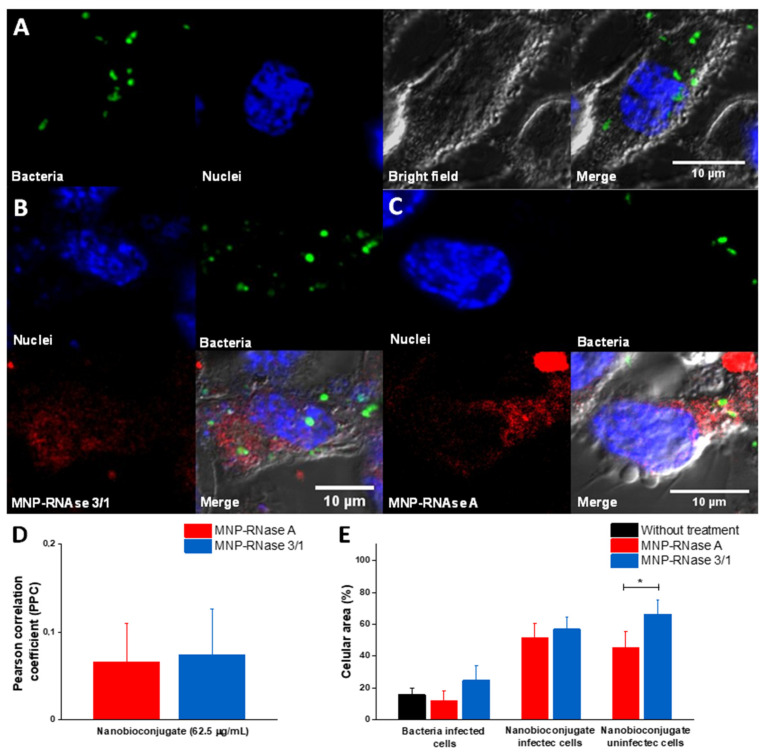
Confocal analysis of infected macrophages with *P. aeruginosa* and exposed to the MBC concentration of nanobioconjugates. (**A**) Control of infected macrophages in the absence of nanobioconjugates. (**B**) Infected macrophages exposed to MNPs-RNase 3/1. (**C**) Infected macrophages exposed to MNPs-RNase A. (**D**) Colocalization analysis between bacteria and nanobioconjugates (*n* = 10 cells). (**E**) Percentage of the cellular area occupied by bacteria or nanobioconjugates for both infected and uninfected cells (*n* = 10 cells * *p*-value < 0.05).

To study the interactions of MNPs-RNase nanobioconjugates and *P. aeruginosa* at the intracellular level, infected macrophages were exposed for 1 h to labeled nanoconjugates at a concentration of 62.5 µg/mL (90% effective intracellular treatment concentration of MNP-RNase 3/1). Toward this end, *P. aeruginosa* bacterial cells were labeled with green DND-26, nanobioconjugates with Rhodamine B and Hoechst dye was utilized to label the macrophage nuclei. Figure 8A shows that prior to exposure to the nanobioconjugates, infected macrophages exhibited a normal elongated morphology without visible cytotoxic effects, which is typical of live cells. Figure 8B,C show infected macrophages after exposure to the MNPs-RNase nanobioconjugates. A colocalization analysis revealed PCC values of approximately 10%, which confirmed that the presence of the nanobioconjugates might lead to inhibition of bacterial growth, due to the contact between the bacteria and the nanobioconjugates. To validate this finding, we calculated the ratio of cell area covered by bacteria and MNPs-RNase nanobioconjugates for both infected and uninfected cells. Figure 8E shows that the surface coverage is independent of the presence of bacteria for both nanobioconjugates and only change as a result of the lower internalization capacity of MNPs-RNase A in uninfected cells (Figure 6C). Furthermore, the coverage of bacteria is virtually the same for infected cells in the presence or absence of the MNPs-RNases. The slight increase in colocalization with bacteria for MNPs-RNase 3/1 (6% vs. 7.4%) in contrast with MNPs-RNase A can be due to the affinity of RNase 3/1 for LPS [15] (Figure 8E). In this regard, Figure S2 shows how the MNPs-RNase 3/1 nanobioconjugates preferentially colocalized closer to bacteria, instead of other intracellular regions. In contrast, the integrated density of MNPs-RNase A is lower in regions where bacteria are predominant.

## 4. Discussion

The intracellular phase of *P. aeruginosa* is one of the most relevant forms of bacterial resistance, which has been considered responsible for the increased pathogenicity of this Gram-negative microorganism [76]. In this regard, it is well-known that this type of intracellular resistance might be involved in the large number of diseases caused by *P. aeruginosa* [76,83]. Thus far, treatments with the ability to combat such intracellular infections appear rather scarce [84]. As a result, there is an urgent need to develop more effective treatments to avoid the intracellular growth of pathogenic microorganisms. Previously, our research group designed and explored the mechanism of action of a novel hybrid ribonuclease RNase 3/1, towards Gram-negative microorganisms [15]. Additionally, numerous studies have demonstrated the effective activity of the human RNase 3 to inhibit the intracellular growth of several microorganisms, including *Mycobacterium* [16] and even the proliferation of biofilm structures of *P. aeruginosa* [12]. In parallel, we have developed a nanoplatform based on modified magnetite nanoparticles (MNPs) for the efficient immobilization of antimicrobial and cell-penetrating molecules. Accordingly, here, we aimed at exploring the potential of combining these two technologies (i.e., MNPs and RNases) to produce more potent treatments for intracellular infections of *P. aeruginosa*.

We designed our MNPs-RNase 3/1 and MNPs-RNase A nanobioconjugates by conjugation of the protein molecules on functionalized MNPs. Once the MNPs were synthesized, the first functionalization step was the conjugation of the organosilane molecule APTES. This step was to render reactivated free amine groups on the surface to facilitate the subsequent conjugation of a heterobifunctional polymeric surface spacer. Here, we conjugated the commercially available polyether amine (PEA), Jeffamine M600, which was previously oxidized to form free carboxyl groups with the aid of KMnO_4_ (details of the oxidation protocol can be consulted elsewhere) [33]. The oxidized polymer contained free carboxyl groups and an amine terminal. This polymeric spacer had the purpose of facilitating membrane translocation and helped to avoid possible detrimental conformational changes on the structure of the RNases after conjugation [85]. Conjugation of PEA to the silanized MNPs proceeded with the aid of glutaraldehyde by forming an imine bond between the amine groups on the surface of the MNPs and the amine terminal of PEA. This was followed by conjugation of the carboxyl terminal of PEA to the N-terminal of the RNase proteins by forming amide bonds. Effective functionalization was verified with the aid of FTIR and TGA (Figure 1C,D). This is critical, as functionalization largely determines the mechanisms of cellular uptake [7,64]. To evaluate whether the magnetite–protein crosslinking could influence the enzymatic activity, the activity of MNPs-RNase 3/1 and MNPs-RNase A was measured using a yeast RNA–agarose plate assay. As shown in Figure 2, the bright area intensity of RNase A and RNase 3/1 was compared with that of the MNPs-RNase A and MNPs-RNase 3/1. Free ribonucleases exhibited 100% catalytic activity compared with MNPs-RNase A and MNPs-RNase 3/1, which still maintained an initial activity of around 15% and 40%, respectively. Although the catalytic activity of the protein was reduced, the antibacterial activity was maintained and increased after immobilization on MNP. Recent studies have demonstrated that indeed, the antimicrobial and catalytic activities appear unrelated [16,86]. Finally, it should be noted that the obtained nanobioconjugates were prepared at a single RNases:MNPs ratio, as detailed in the Materials and Methods section. The surface coverage can be eventually optimized to attempt higher antimicrobial activity levels without compromising biocompatibility.

To evaluate the potential translational significance of our results in an in vivo scenario, the cytotoxic effect was determined in mammalian cells. The results showed no alteration of the membrane integrity, even at the highest concentration of MNPs-RNases assayed (100 µg/mL). Regarding the antibacterial action of the proteins and nanobioconjugates, the extracellular activity toward *P. aeruginosa* showed a range of inhibitory activity above 31µg/mL for the hybrid 3/1 but no effect for RNase A (data not shown). To gain further insight into the antimicrobial effect of both proteins after immobilization, we determined the bactericidal activity. Surprisingly, antimicrobial activity was observed in RNase A after the functionalization process (Figure 4). Additionally, we observed antimicrobial action for bare MNP. This result suggests that the conjugation process might enhance the antimicrobial activity of the RNases. In particular, the concentration of immobilized RNase 3/1 needed to achieve 90% of extracellular antimicrobial activity is 3.47 µg/mL, which is only 11% of the amount required when using free protein. We hypothesize that by forming the bionanoconjugate, proteins tend to cluster on the surface of the MNPs, which in turn, leads to an enhanced ability to promote destabilization of the bacterial envelope. Moreover, recent reports have demonstrated that Iron Oxide Nanoparticles (IONPs) show antimicrobial activity at relatively high concentrations. The activity can be further improved by changing the surface potential and accessible surface functional groups. These changes aim at altering the nano–bio interface by enhancing the production of ROS [87]. Furthermore, the superior antibacterial activity of the MNPs-RNase nanobioconjugates is evident when compared with those recently prepared by us through conjugation of BUF-II on MNPs (MNPs-BUF-II). This is considering that an inhibition of 50% of bacterial growth is achieved with 1 mg/mL of MNP-BUF-II nanobioconjugates [33], which is 66 times higher than that of MNP-RNase 3/1 to reach 90% growth inhibition.

RNases are present in all life kingdoms, from viruses to vertebrates, where their main role is related to RNA degradation. The RNase A superfamily is particularly attractive, since it is vertebrate-specific and offers a wide range of potential therapeutic activities, such as antitumoral, antiproliferative, immunosuppressive and antipathogenic [61,88]. Interestingly, the human members of the family show different cell type distributions and roles in the human immune system [9]. Structural-functional studies identified a region at the N-terminus that encompasses most of the protein antimicrobial activity [89,90]. To note, most of the family members can be inhibited inside the mammalian cell cytosol due to the presence of a Ribonuclease Inhibitor (RI) that protects the host cell from the potential RNase cytotoxic activity [91,92,93]. The RI binds at a 1:1 ratio with an unusual high affinity to all tested human RNases (at the nM–pM range) and avoids any protein toxic effect at the cell cytoplasm. Nevertheless, RI protection for the host cell can be overcome by protein engineering by means of conjugation to nanomaterials or oligomerization [46,91,94,95]. This strategy has, indeed, been proposed for targeting cancer cells and other chemotherapies [46,65,96]. However, one of the most common and challenging obstacles to achieve a full cytotoxic effect is insufficient cellular uptake [46,85]. To overcome this issue, RNases can be immobilized on MNPs, which have been demonstrated to enhance the penetration potency of various proteins. Moreover, they are considered safe due to their reduced hemolytic, platelet aggregation and cytotoxic effects. This was confirmed in the present study and agrees well with previous studies of our group [33,34,97].

Based on the promising results, the next aim was to explore the intracellular effect of the engineered nanodelivery system against the intracellular growth of *P. aeruginosa* PAO1, as a strategy to reduce or eradicate its intracellular growth. The present study focused only on this strain but can be easily extended to other strains of clinical importance. The choice of a unique, well-characterized strain was essential for the development and validation of the model. Once the bacteria phagocytosis process takes place by THP-1 cells differentiating into macrophages, the intracellular *P. aeruginosa* remains confined within vacuoles with active replication for at least 24 h [25]. Our results confirmed an appropriate bacterial internalization into macrophages (Figure 8). Regarding nanobioconjugate internalization, previous studies demonstrated membrane translocation without causing significant membrane disruption [33]. A wide variety of strategies for endosomal escape have been enabled by different inorganic nanoparticle supports, which have been also confirmed for MNPs. Notwithstanding, in the present case, we observe a tendency to endosomal entrapment, which could protect cells from potential RNase cytotoxicity and might be promoted by the biological natural secretory routing of RNase 3 [78]. Confocal microscopy images of THP-1 cells incubated with the MNP-RNases showed punctate fluorescence, thereby suggesting endosomal entrapment, a characteristic feature of phagocytic cells such as the macrophages [98]. The confocal images obtained showed different levels of intracellular clustering and localization, which suggest different types of internalization mechanisms for the MNP-RNases. This is in line with recent reports, which indicate that internalization pathways might vary depending on the size of the nanovehicles or the presence of particle clusters [82]. Additionally, the percentage of colocalization of MNPs-RNases within lysosomes is closer to 50%, a fact that in part, might explain the low cytotoxicity of the MNPs-RNases. In this regard, it has been reported that some ribonucleases can become highly cytotoxic [91,96] at concentrations around 5 to 30 µM [99]; however, the highest concentration evaluated was only 1.6 µM (immobilized protein), where only 50% was estimated to reach the cytoplasm (0.8 μM). Likewise, since half of the MNPs-RNases accumulate into lysosomal compartments where phagocytized *P. aeruginosa* tends to accumulate [21], it is reasonable that a two-fold concentration of MNP-RNase 3/1 is needed to reach an equivalent antibacterial activity. This could also explain why the effective intracellular treatment concentration is higher than the effective extracellular treatment concentration for the MNPs-RNase 3/1. We are planning future studies to study in detail the intracellular trafficking and fate of the MNPs-RNases into infected THP-1 cells. Moreover, we envisage to test other infection models, such as the dreaded mycobacteria, which are particularly resistant to most therapies and therefore, are likely to be susceptible to a specifically engineered nanodelivery system based on MNPs.

## 5. Conclusions

The fight against intracellular infection by the pathogen *P. aeruginosa* has become one of the main challenges at the medical level due to its multiple mechanisms of pharmacological resistance. In the search for new treatment alternatives against this infection, the antimicrobial members of the Ribonuclease family (RNases), which naturally participate in the prevention of infection within our biological fluids, have been considered an attractive alternative. One of the main challenges is to assure that the RNases reach the intracellular space at sufficiently high concentrations to effectively reduce the viability of the pathogen. Here, we overcome such issue by immobilizing the RNase 3/1 hybrid protein on magnetite nanoparticles (MNPs). This recombinant fusion protein combines the high catalytic activity of human RNase 1 with the particular antimicrobial properties of RNase 3. Additionally, the same immobilization protocol was conducted for the RNase A as a control. The obtained nanobioconjugates (i.e., MNPs-RNase 3/1 and MNPs-RNase A) demonstrated a higher efficacy to reduce the viability of *P. aeruginosa* both extra- and intra-cellularly in macrophages at concentrations lower than those obtained previously by us with BUF-II nanobioconjugates. This is attractive from the potential profit viewpoint (i.e., higher margins) as lower amounts of the costly RNases might be required to achieve the same level of inhibition. In the intracellular case, this was achieved by internalization percentages above 20% and 60% for MNPs-RNase 3/1 and MNPs-RNase A, respectively. Moreover, about 50% of the nanobioconjugates locate within lysosomes, which are the compartments where the pathogens are trapped. Internalization appears to proceed by different mechanisms, which will be studied in an upcoming contribution. Despite superior antibacterial activity, the nanobioconjugates showed non-significant hemolytic and platelet aggregation activities as well as significantly low cytotoxicity, which make them safe and appealing in the eventual situation of in vivo studies. Moreover, we expect to continue exploring cell internalization and trafficking mechanisms of our nanobioconjugates and their relation with their antimicrobial action. Additionally, due to the magnetic responsiveness of magnetite, further studies in regard to applying uniform or oscillating magnetic fields upon delivery will be valuable to evaluate whether antimicrobial action can be increased even further. The developed nanoplatform holds promise for more effective and potentially profitable therapies for the currently emerging resistant microorganisms with the ability to infect cells intracellularly, which nowadays is a very concerning health issue of global interest.

## Supplementary Materials

The following are available online at https://www.mdpi.com/1999-4923/12/7/631/s1, Figure S1. Total protein quantification (BCA assay) upon immobilization of both RNases on MNPs. We used 500 µg/mL of nanobioconjugates and established a percentage of 22.77% of RNase 3/1 and 23.19% of RNase A on in the surface of the nanobioconjugates [*n* = 3]. Figure S2. Normalized integrated density of nanobioconjugates on different cellular regions with respect to the distance to bacteria. Each value was normalized respect to the integrated density of nanobioconjugates colocalized with bacteria [same: nanobioconjugate colocalized with bacteria, close: nanobioconjugates located near to bacteria (less than 1 µm) and distant: nanobioconjugates located ≥2 µm from bacteria]. Significant differences between locations and MNP-RNases were estimated with the Two-Way ANOVA and Dunnett tests. * *p*-value < 0.05 (*n* = 10 bacteria inside the cells).
