# Supplementary material for: Magnetite Nanoparticles Functionalized with RNases against Intracellular Infection of Pseudomonas aeruginosa
Source: Pharmaceutics. 2020 Jul 6;12(7):631. doi: 10.3390/pharmaceutics12070631 (PMC7408537; doi:10.3390/pharmaceutics12070631)
Supplement: Supplementary file 1 [file pharmaceutics-12-00631-s001.pdf]

pharmaceutics

Supplementary Materials: Magnetite Nanoparticles Functionalized with RNases against Intracellular Infection of *Pseudomonas aeruginosa*

Nathaly Rangel-Muñoz, Alejandra Suarez-Arnedo, Raúl Anguita, Guillem Prats-Ejarque, Johann F. Osma, Carolina Muñoz-Camargo, Ester Boix, Juan C. Cruz and Vivian A. Salazar

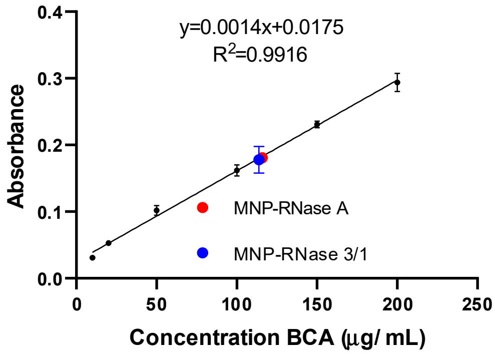

Figure S1. Total protein quantification (BCA assay) upon immobilization of both RNases on MNPs. We used 500 µg/mL of nanobioconjugates and established a percentage of 22.77% of RNase 3/1 and 23.19% of RNase A on the surface of the nanobioconjugates [*n* = 3].

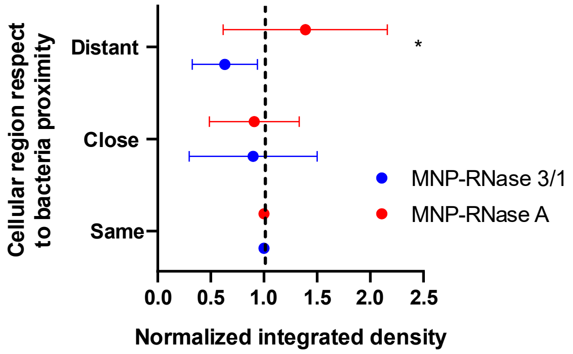

Figure S2. Normalized integrated density of nanobioconjugates on different cellular regions with respect to the distance to bacteria. Each value was normalized respect to the integrated density of nanobioconjugates colocalized with bacteria [same: nanobioconjugate colocalized with bacteria, close: nanobioconjugates located near to bacteria (less than 1 µm) and distant: nanobioconjugates located ≥2 µm from bacteria]. Significant differences between locations and MNP-RNases were estimated with the Two-Way ANOVA and Dunnett tests. * p-value < 0.05 (n = 10 bacteria inside the cells).
